# Evaluation of Retentive Force and Long-Term Performance of 4 Mol% Yttria Partially Stabilized Zirconia (4Y-PSZ) Double Crowns

**DOI:** 10.7759/cureus.75705

**Published:** 2024-12-14

**Authors:** Mizuma Yoshizawa, Masayuki Takaba, Takahito Osawa, Fuminori Iwasa, Kazuyoshi Baba

**Affiliations:** 1 Department of Prosthodontics, Graduate School of Dentistry, Showa University, Tokyo, JPN; 2 Division of Fixed Prosthodontics, Department of Restorative and Biomaterials Sciences, Meikai University School of Dentistry, Saitama, JPN

**Keywords:** 4y-psz, cad/cam, computer-aided design and computer-aided manufacturing, double crown, long-term performance, zirconia

## Abstract

Purpose: This study aims to evaluate the effects of taper angle and the number of insertion-removal cycles on the retention force of 4 mol% yttria partially stabilized zirconia (4Y-PSZ) double crowns over time.

Materials and methods: Primary and secondary crowns were fabricated using 4Y-PSZ with taper angles of 2°, 4°, and 6° (n=15). Retention force during crown removal was measured after applying 50-N and 100-N loads. Insertion-removal cycles were performed at 0, 2500, 5000, 7500, and 10,000 cycles, and retention force was assessed at each stage. Contact conditions between the inner and outer crowns were evaluated via color mapping at 0 and 10,000 cycles. Two-way ANOVA was used for statistical analysis, followed by Tukey's test for multiple comparisons.

Results: Retention force significantly decreased with smaller taper angles and higher cycle numbers (p<0.01). For a taper angle of 4°, the initial retention force of 10.36 N decreased significantly to 5.98 N after 10,000 cycles (p<0.01). For a taper angle of 6°, the initial retention force of 1.64 N dropped to 0.03 N after 10,000 cycles. Color mapping revealed continuous contact along the axial surface, with strong point contacts, particularly in the central axial region. Smaller taper angles exhibited stronger contact points, which decreased with repeated cycles, indicating changes in contact conditions.

Conclusions: Higher retentive forces were achieved with increased loading pressures and smaller taper angles, while repeated insertion-removal cycles led to a progressive reduction in retention. These findings provide foundational data supporting the clinical application of 4Y-PSZ double crowns.

## Introduction

Double crowns are retention devices widely used in removable partial dentures, offering significant advantages in the distribution of occlusal forces. These crowns efficiently transmit forces along the axis of the abutment teeth, thereby optimizing load distribution during mastication [1,2]. Compared to conventional clasps, double crowns improve hygiene management for abutment teeth and superior esthetics [3]. As a result, they are applied to both natural teeth and implants, demonstrating excellent survival rates for both abutments [4,5]. Traditionally, double crowns are fabricated from precious metals, such as gold alloys, requiring a composite resin layer on the surface to enhance esthetics. However, using such metals is costly and carries the risk of metal allergies [6]. Additionally, the manufacturing process involves the labor-intensive lost-wax technique, necessitating a high level of technical skill to achieve precise adjustments between the primary and secondary crowns [7,8].

In contrast, zirconia has emerged as a promising alternative material for dental restorations owing to its excellent properties, including biocompatibility, mechanical strength, chemical resistance, and superior esthetics. Moreover, zirconia's market price is more stable than gold alloys. Zirconia-based prostheses are fabricated using computer-aided design (CAD)/computer-aided manufacturing (CAM) systems, enabling the digital design and production of crowns while minimizing technical errors during manufacturing [9,10].

Various types of zirconia are available, each with distinct mechanical properties and translucencies. First-generation zirconia, commonly composed of 3 mol% yttria-stabilized tetragonal zirconia polycrystal (3Y-TZP), exhibits high mechanical strength but limited translucency. As a result, porcelain layering is often required to achieve aesthetic outcomes. However, advancements in zirconia technology have led to the development of high-translucency zirconia containing 4-6 mol% yttria (yttria partially stabilized zirconia: Y-PSZ), which can be used for anterior crowns without porcelain layering and may be suitable for double crowns.

Previous studies have shown that the retentive force of double crowns made from dental alloys depends on the taper angle of the primary crown and the applied load, with smaller taper angles and higher applied loads yielding greater retentive forces [11-13]. Despite these findings, research on the retentive characteristics of zirconia double crowns remains limited [9,14,15].

This study aimed to investigate the retentive characteristics of 4 mol% yttria partially stabilized zirconia (4Y-PSZ) double crowns by examining (1) the effect of the taper angle of the primary crown and the applied load on the retentive force and (2) the long-term stability of retentive forces after repeated insertion and removal cycles.

## Materials and methods

Manufacture of double crowns

**Figure 1:**
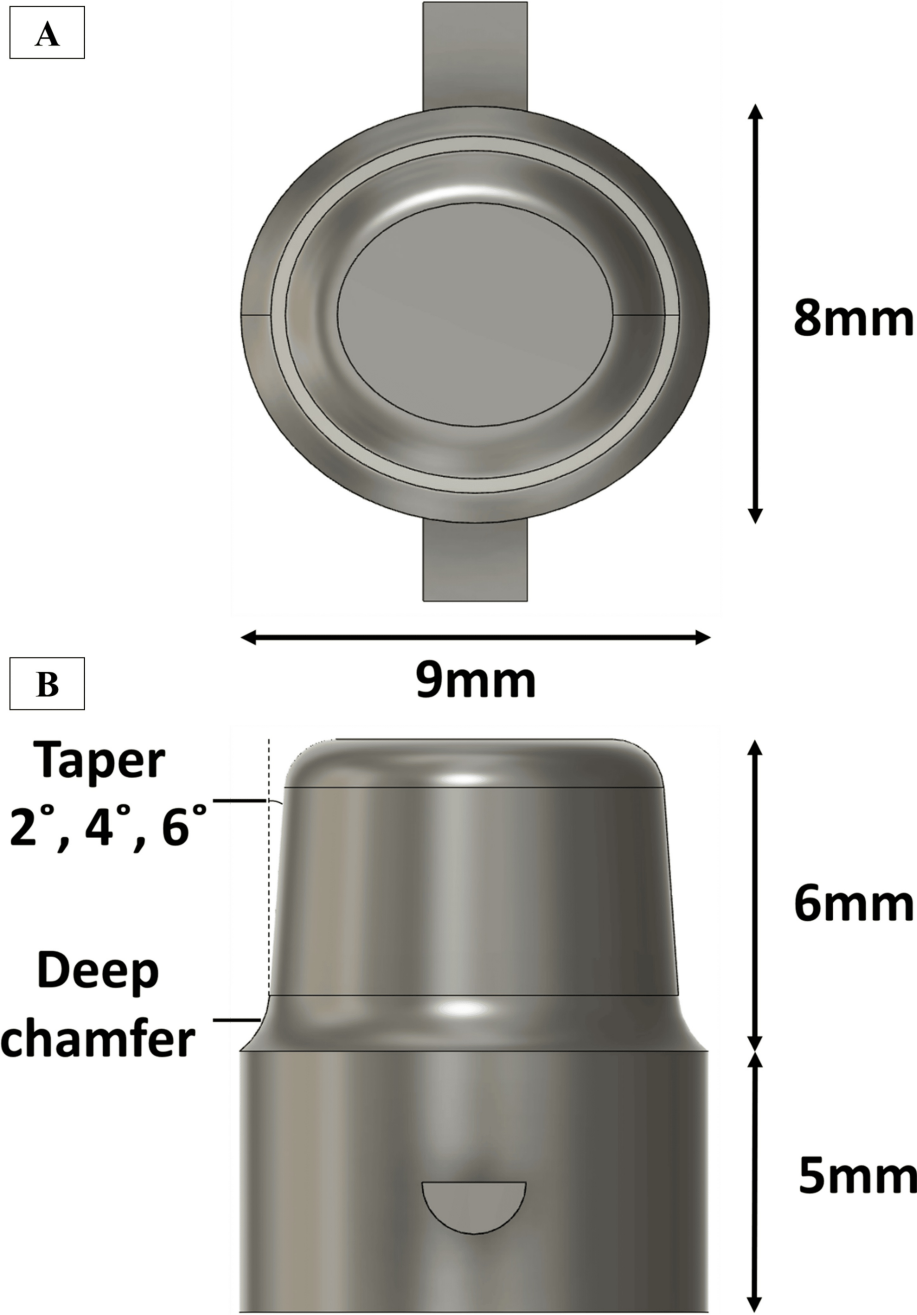
Design of the primary crown fabricated using 3D CAD software A: Top view of the primary crown. B: Side view of the primary crown. 3D: three-dimensional, CAD: computer-aided design

Both primary and secondary crowns were fabricated using 4Y-PSZ discs (LuxenE2, GeoMedi, Seoul, Korea). The primary crown was designed as a truncated cone with a major diameter of 9 mm, a minor diameter of 8 mm at the cervical margin, and a height of 6 mm. Using three-dimensional (3D) CAD software (Fusion 360, Autodesk, San Francisco, CA, USA), primary crowns were modeled with taper angles of 2°, 4°, and 6°, a 1-mm-deep chamfer, and a curvature radius of 0.8 mm. A semi-cylindrical knob (1.5 or 2 mm) was incorporated into the axial surface of the primary crown to facilitate the mounting of the secondary crown and to serve as a landmark during retentive force measurements (Figure 1).

**Figure 2:**
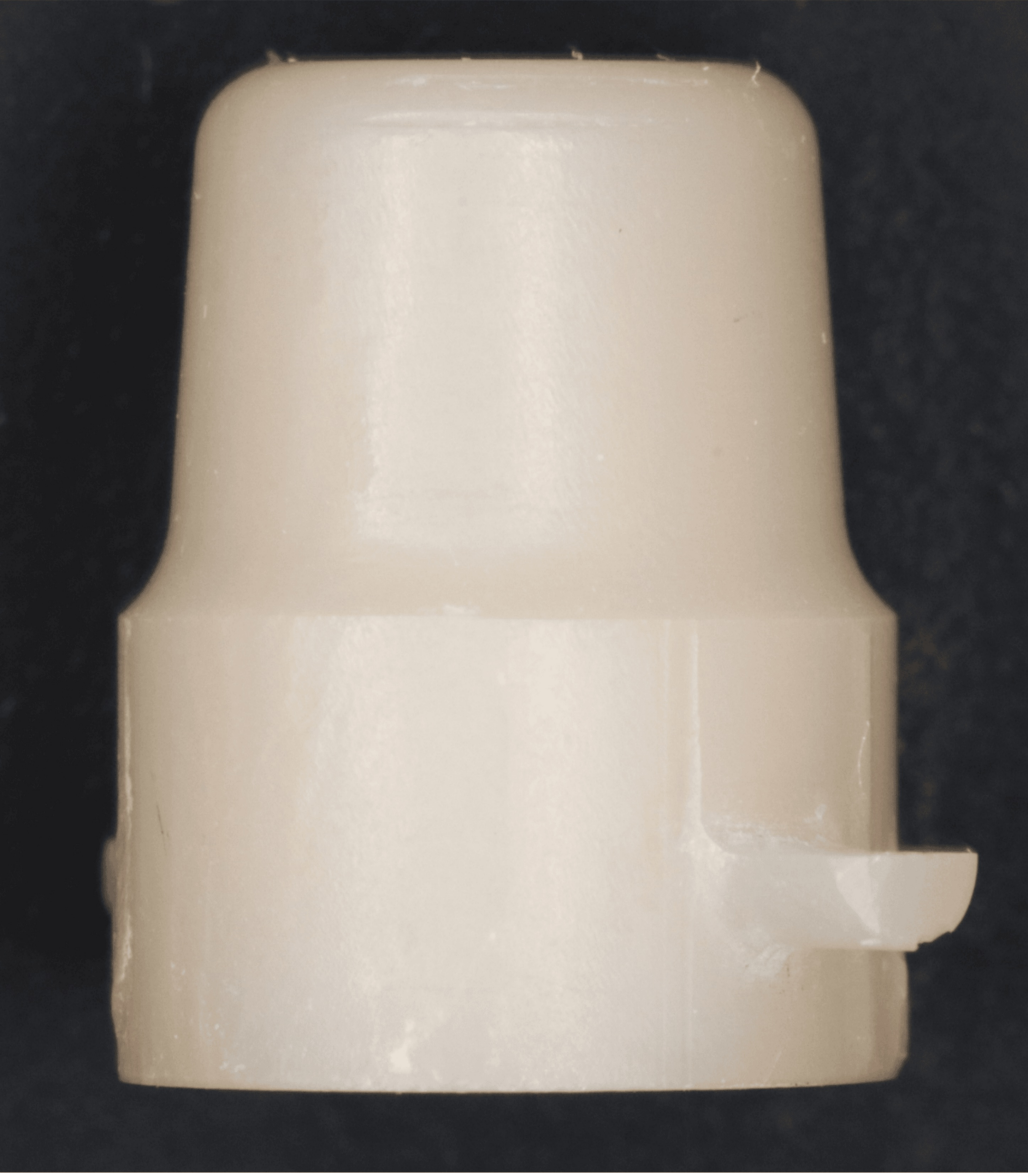
Primary crown fabricated using 4Y-PSZ discs 4Y-PSZ: 4 mol% yttria partially stabilized zirconia

The primary crowns were machined using a five-axis milling machine (DWX-52D, DGSHAPE, Shizuoka, Japan). Following the manufacturer's guidelines, the crowns were sintered in a furnace (ZYRCOMAT 6000, VITA, Bad Säckingen, Germany) at 1,450°C for two hours, with a total sintering process duration of approximately five hours (Figure 2).

To design the secondary crowns, STL data of the sintered primary crowns were acquired using a 3D scanner (E4, 3Shape, Copenhagen, Denmark). Secondary crowns were then modeled with CAD software (Dental Manager, 3Shape), ensuring a minimum wall thickness of 1 mm, no cement space on the axial surface, and a 0.3-mm thickness on the occlusal surface. Knobs of varying lengths were added to the lateral surface to serve as landmarks for retentive force measurement and superimposition.

**Figure 3:**
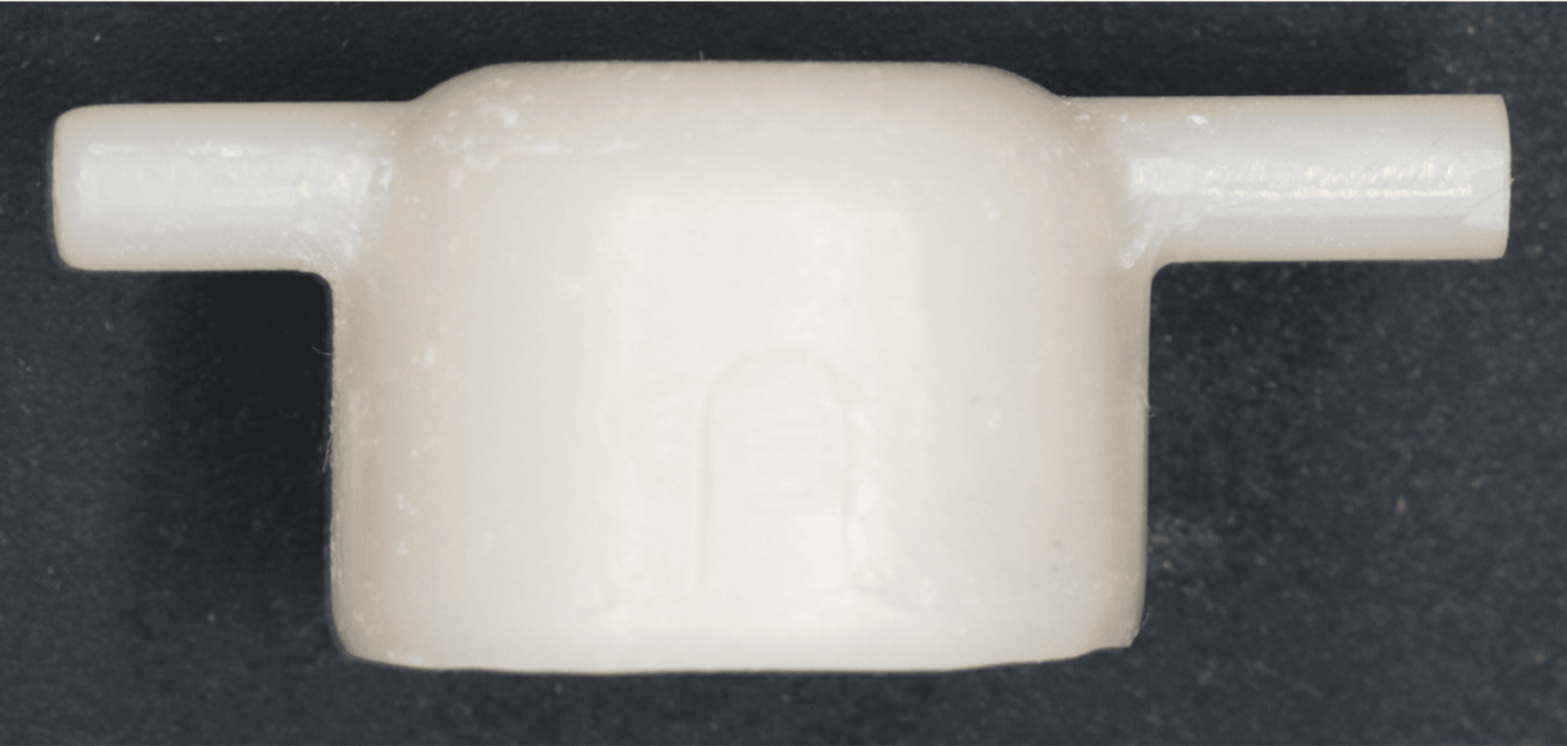
Secondary crown fabricated using 4Y-PSZ discs 4Y-PSZ: 4 mol% yttria partially stabilized zirconia

Secondary crowns were fabricated using the same milling and sintering methods as the primary crowns. For each taper angle, five pairs of primary and secondary crowns were fabricated, resulting in a total of 15 double-crown sets (Figure 3).

Measurement of retentive force

**Figure 4:**
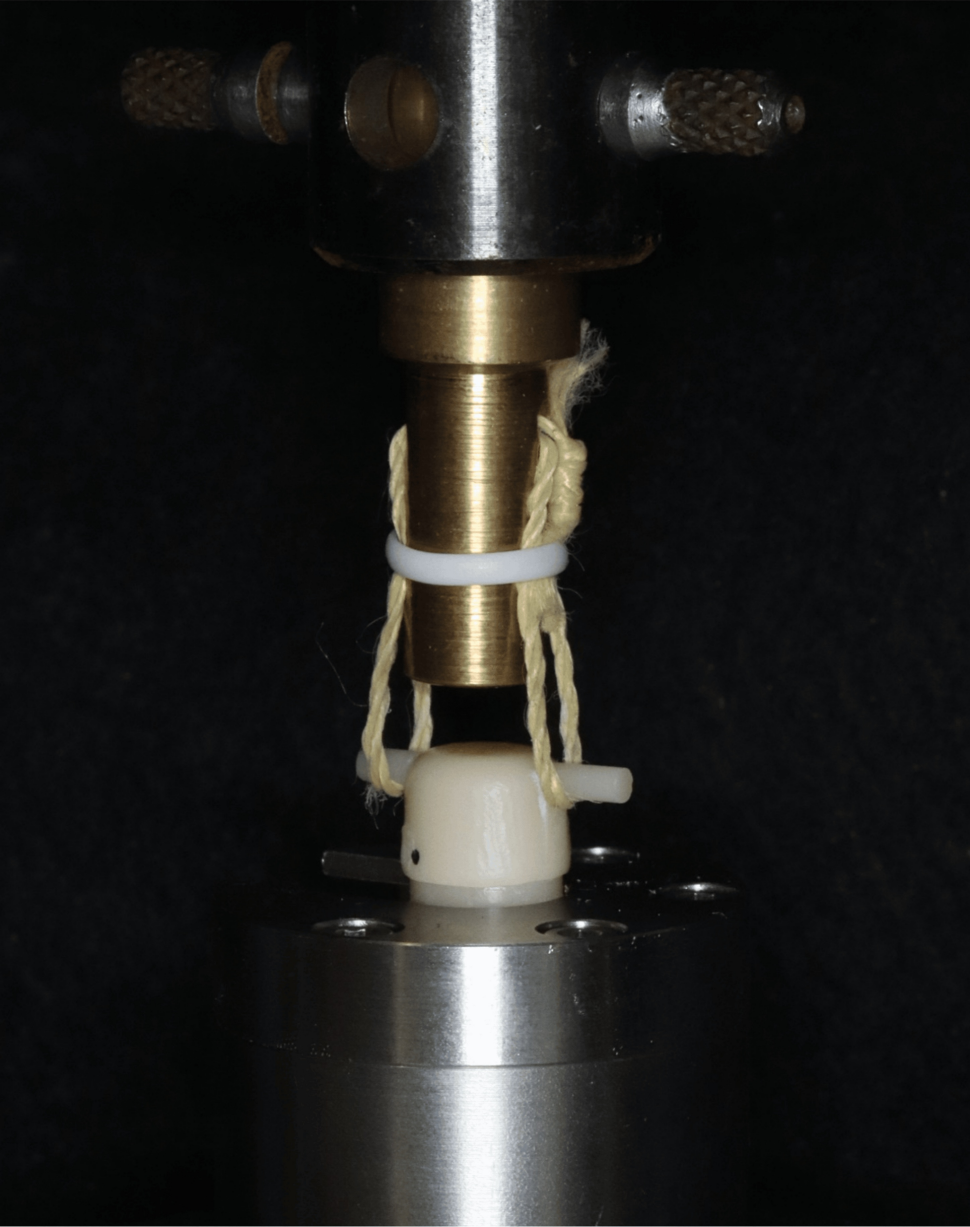
Retentive force measurement of double crowns using a tensile testing machine

After the underside of the primary crown was fixed to a tensile testing machine (5500R, Instron, Norwood, MA, USA) fixture using a self-curing resin (UNIFAST III, GC, Tokyo, Japan), the secondary crown was set on the primary crown, and loads of 50 and 100 N were applied for five seconds. The retentive force was measured by removing the secondary crown vertically using a Kevlar cord at a crosshead speed of 40 mm/min (Figure 4). The maximum value measured was recorded as the retentive force. Measurements were performed five times, and the mean values were calculated.

**Figure 5:**
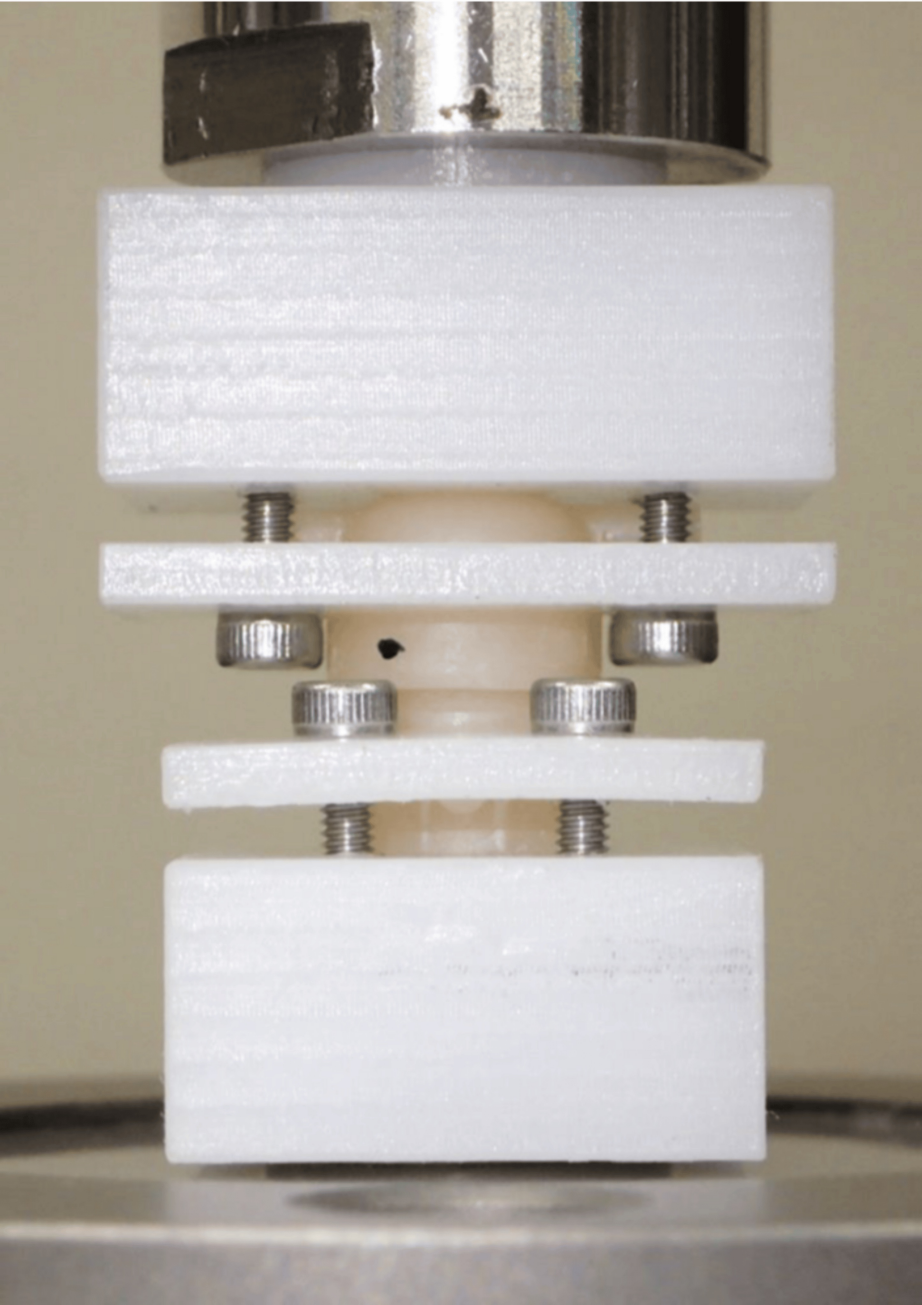
Insertion and removal tests of double crowns using a fatigue testing machine

Repeated insertion and removal tests were performed for 10,000 cycles for each crown at a cyclic load of 50 N using a dynamic fatigue testing machine (E3000, Instron) (Figure 5). The frequency was set to approximately 0.5 Hz, and the amplitude was set to approximately 4 mm. Retentive forces were measured using a tensile testing machine (5500R, Instron) after every 2,500 insertion-and-removal cycles. The secondary crown was set on the primary crown, and a 50-N load was applied to the secondary crown for 5 seconds. The retentive force was measured five times using the method described previously, and the average of the five values was calculated.

Observation of the contact surfaces between primary and secondary crowns using color mapping

A scanning spray (CLEASCAN, BSA Sakurai, Nagoya, Japan) was evenly applied to both crowns to assess the contact surfaces between the primary and secondary crowns. STL data for the primary crown, secondary crown, and their engaged state were acquired using a pattern projection-type 3D digitizer (FLARE Pro 16M, TTS, Tokyo, Japan).

The engaged state of the crowns was analyzed after 0 and 10,000 cycles of insertion and removal under a 50-N load. Changes in surface conditions for crowns with taper angles of 2°, 4°, and 6° were visualized using color mapping techniques. Data superimposition was performed following a triple-scan protocol [16].

Observation of the primary crown surface

Surface condition changes in the contact areas of the primary crown were observed before and after 10,000 cycles of insertion and removal for crowns with taper angles of 2°, 4°, and 6°. Observations were conducted using a scanning electron microscope (SEM) (Flex SEM 1000, Hitachi, Tokyo, Japan).

Statistical analysis

A two-way analysis of variance (ANOVA) was performed to evaluate the retentive force, with loading forces (50 and 100 N) and taper angles (2°, 4°, and 6°) as the two factors. The significance level was set at 1%. Tukey's honestly significant difference (HSD) test was used to compare multiple groups if significant differences were found.

Another two-way ANOVA was conducted to analyze retentive force based on the number of insertion-removal cycles after fatigue testing and taper angles (2°, 4°, and 6°). The significance level was also set at 1%. When significant factors were identified, Tukey's HSD test was performed for multiple comparisons.

## Results

Retentive force measurement results

**Figure 6:**
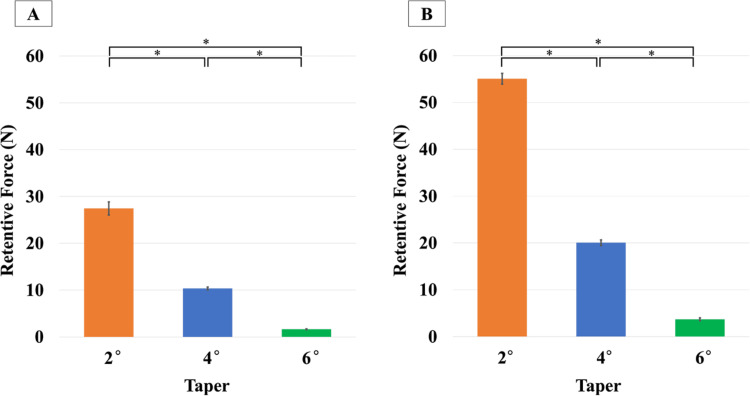
Retention force for each taper under 50-N and 100-N loads A: 50-N load. B: 100-N load. The data is presented as mean. The whiskers represent the standard deviations. A two-way analysis of variance (ANOVA) was performed to evaluate the retentive force, with loading forces (50 and 100 N) and taper angles (2°, 4°, and 6°) as the two factors. The significance level was set at 0.01. * p<0.01.

Figure 6 shows the retentive force measurements after applying a load of 50 or 100 N for five seconds with the primary crown in place. Two-way ANOVA and Tukey's test revealed significant differences across all combinations.

**Figure 7:**
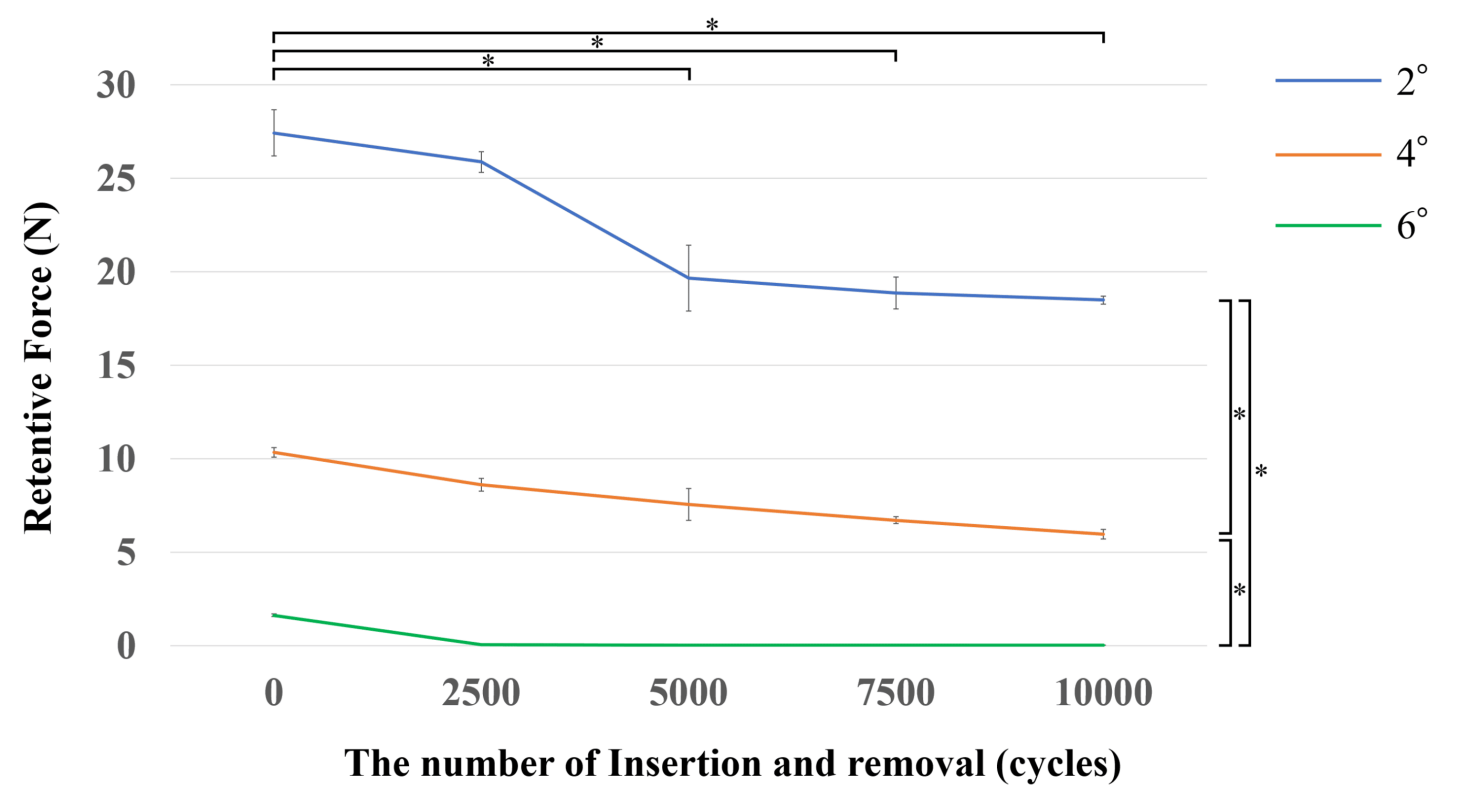
Changes in retentive force after fatigue test The data is presented as mean. The whiskers represent the standard deviations. A two-way analysis of variance (ANOVA) was performed to evaluate the retentive force, with the number of insertion-removal cycles and taper angles (2°, 4°, and 6°) as the two factors. The significance level was set at 0.01. * p<0.01.

The changes in retentive force under a 50 N load are presented in Figure 7. The initial retentive forces were 20.8 N for the 2° taper, 8.5 N for the 4° taper, and 1.6 N for the 6° taper. After 10,000 insertion-and-removal cycles, the retentive force decreased to 18.5 N (−32.6%) for the 2° taper, 6.0 N (−42.3%) for the 4° taper, and 0.3 N (−98.1%) for the 6° taper. A significant decrease in retentive force was observed as the taper angle increased (p<0.01).

Regarding the number of insertion-and-removal cycles, significant reductions in retentive force were observed for the 2° and 4° tapers across all combinations, except for comparisons between 0 and 2,500 cycles (p<0.01). In contrast, no significant differences were found for the 6° taper across the cycles.

Color mapping of the primary and secondary crown contact areas

**Figure 8:**
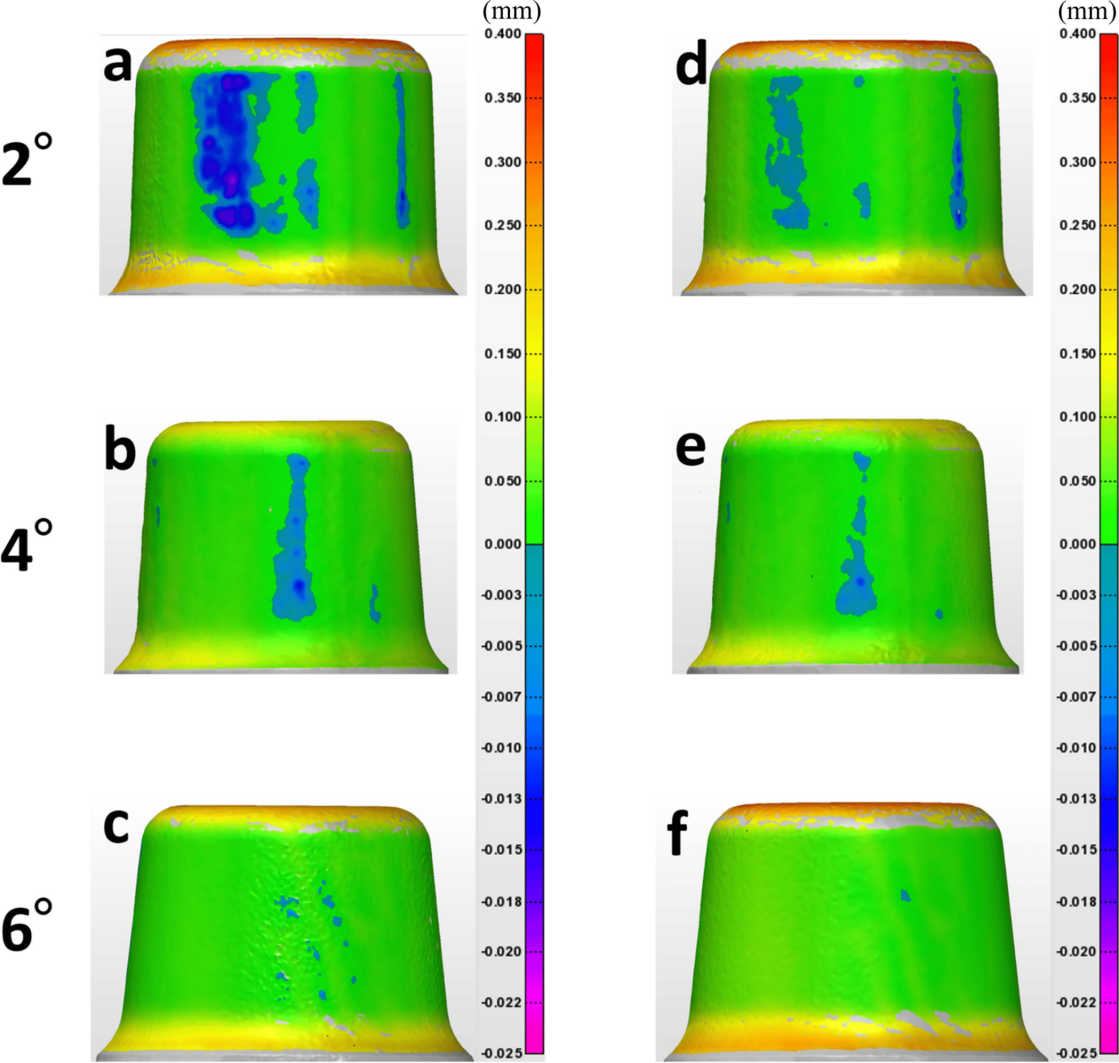
Color mapping visualizing the contact area of primary crowns before and after 10,000 insertion-removal cycles a-c: Before the insertion-removal cycles at each taper. d-f: After 10,000 insertion-removal cycles at each taper. The color bar indicates the clearance between the primary and secondary crowns on a millimeter scale.

The internal contact conditions of the primary crown at tapers of 2°, 4°, and 6° before and after 10,000 insertion-and-removal cycles are shown using color mapping in Figure 8. For the 2° and 4° tapers, strong point contacts were observed across the entire axial surface, particularly in the central region, before 10,000 insertion-and-removal cycles. In contrast, such contacts were scarcely observed for the 6° taper.

The upper surface of the primary crown was designed with an approximate clearance of 0.3 mm. The number of strong contact areas increased as the taper angle decreased, and these areas diminished with the increasing number of insertion-and-removal cycles.

Internal contact condition of a double crown

**Figure 9:**
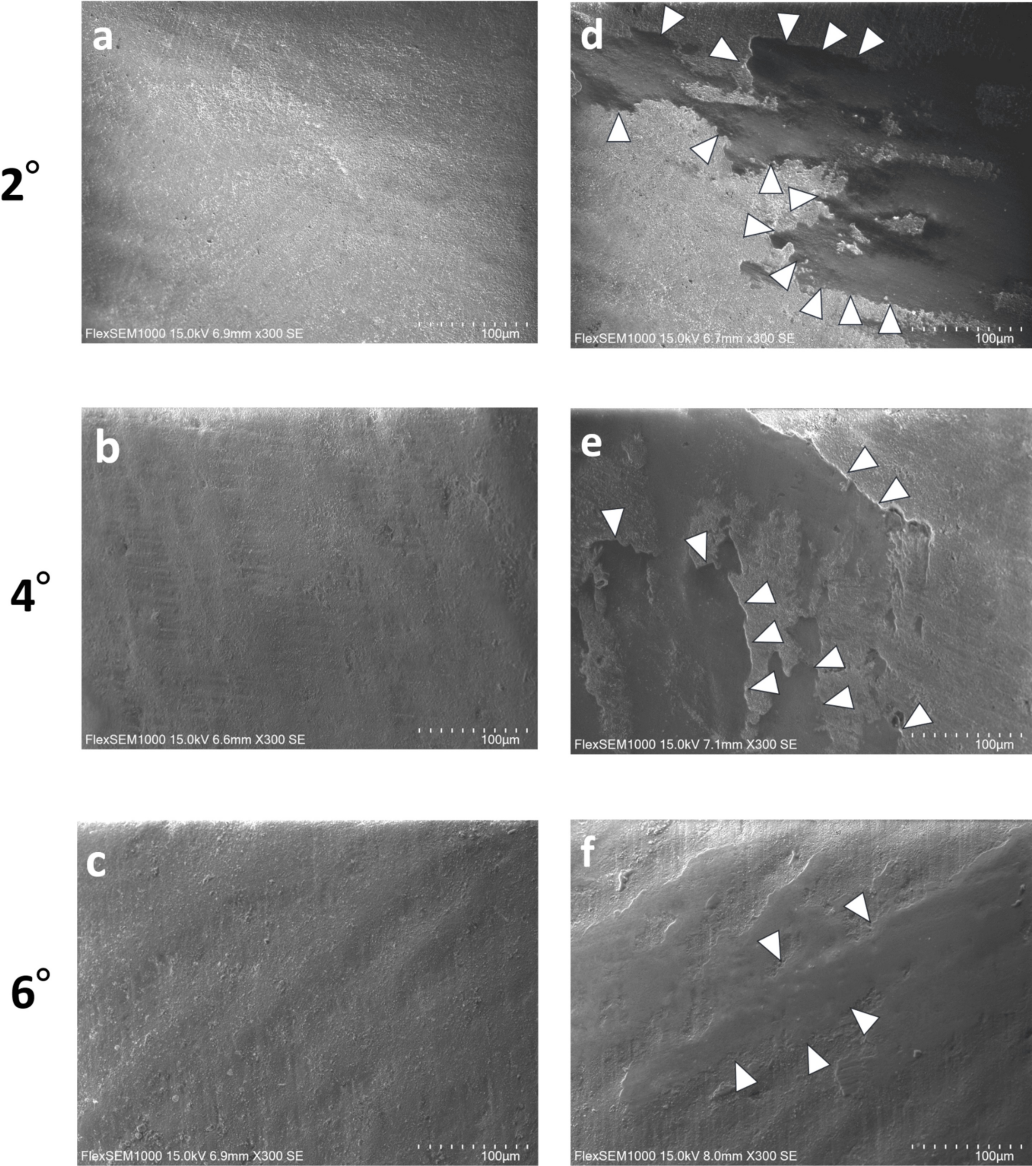
SEM images of the primary crown contact area before and after 10,000 insertion-removal cycles a-c: Before the insertion-removal cycles at each taper. d-f: After 10,000 insertion-removal cycles at each taper. White arrows indicate more worn areas.

Representative SEM images of the primary crown surfaces (2°, 4°, and 6°) before and after 10,000 insertion-and-removal cycles are shown in Figure 9. Compared to pre-test conditions, the primary crown surfaces after 10,000 cycles exhibited a smoother texture resulting from wear and delamination. The worn areas showed a layered structure. The 2° taper exhibited more significant wear than the 4° and 6° tapers, with more pronounced surface changes observed.

## Discussion

Main findings

This study investigated the effects of taper angle, applied load, and insertion-removal cycles on the retentive force of double crowns fabricated from 4Y-PSZ. The results revealed that higher retentive forces were associated with greater loading pressures and smaller taper angles, while retentive force diminished with increasing insertion-removal cycles.

Equipment and conditions

All specimens were fabricated under standardized conditions using CAD/CAM technology, with taper angle being the only variable. The dimensions of the primary crowns (long diameter, 9 mm; short diameter, 8 mm; height, 6 mm) were designed to reflect the average values for standard Japanese molars [17], resulting in an elliptical truncated cone shape. The secondary crowns were designed with a minimum thickness of 1.0 mm to ensure structural integrity, and no fractures were observed during the experiments. In the literature, Nakagawa et al. set the cement space between the primary and secondary crowns at 0 and 10 μm and 30 μm by Schwindling et al. [9,10]. A pilot study was conducted to assess the influence of cement space on retention to optimize retention. Retention tests using initial cement spaces of 0, 10, and 30 μm demonstrated that satisfactory retentive forces could not be achieved with 10 or 30 μm. Consequently, a cement space of 0 μm was selected for this study. No post-sintering polishing was performed to prevent morphological changes in the contact surfaces caused by polishing.

Retentive force

Consistent with previous studies [8,18], this study confirms that taper angle and loading pressure significantly influence the retentive force of double crowns. Additionally, the condition of the contact surfaces deteriorates with repeated insertion and removal cycles [19]. Smaller taper angles and higher loads resulted in greater initial retentive force and a smaller reduction in retention after insertion and removal cycles, aligning with prior research findings.

The average occlusal forces during mastication and maximum bite force have been reported as approximately 38.7 and 131.5 N, respectively, for patients with implant overdentures and 24.0 and 74.6 N, respectively, for patients with complete dentures [20]. Based on these data, retention force testing in this study was conducted under two load conditions (50 and 100 N), while insertion-and-removal cycle testing was performed under a 50-N load.

For gold alloy double crowns, a 5-10 N retentive force has been shown to prevent denture dislocation without causing adverse effects on periodontal tissues [21,22]. In this study, the retentive force of the 2° taper crown decreased from 27.4 to 18.5 N after 10,000 insertion-and-removal cycles, still exceeding the necessary retention threshold. However, excessive retentive force may make insertion and removal challenging for patients. Conversely, the 6° taper crown had an initial retentive force of 1.6 N, which was insufficient for secure retention. While previous studies on platinum-gold alloy double crowns report optimal retention at a 6° taper [22], zirconia's higher elastic modulus reduces vertical displacement during loading, limiting the wedge effect. Consequently, a smaller taper angle is required to retain zirconia double crowns adequately. The 4° taper crown demonstrated a retentive force reduction from 10 to 6 N over 10,000 cycles, maintaining a retention level within the ideal range. Since dentures are removed an average of 2.74 times per day [23], the 10,000 insertion-and-removal cycles simulated approximately 10 years of use. These results suggest that 4Y-PSZ double crowns can provide long-term reliable retention, making them a viable option for clinical application.

Changes in surface contact

The contact areas of the double crowns experienced notable alterations due to repeated insertion and removal cycles. This study verifies these changes using digital technologies. A high-precision industrial non-contact 3D measurement device was employed to convert the surface morphologies of the primary and secondary crowns into STL data.

Color mapping was performed using the triple-scan protocol to overlay the digital models and assess the contact conditions [16]. Unlike traditional microscopy methods, which involve destructive sectioning and are commonly used in vitro to evaluate fit [24], the digital approach enabled a non-invasive examination of micrometer-level changes. By applying thresholds during color mapping, it was possible to comprehensively visualize contact conditions across the entire surface. SEM observations revealed wear on the contact surfaces after the insertion-removal tests. This wear was likely caused by delamination at the crystal level, contributing to a decrease in the retentive force of the double crowns. Specimens with smaller taper angles exhibited more pronounced wear, correlating with higher initial retentive forces and greater mechanical engagement during use. Color mapping corroborated the SEM findings, showing that the wear locations on the surfaces corresponded directly to the contact areas identified during digital analysis. These results illustrate that zirconia double crowns undergo a distinct retention mechanism different from that of metal-based crowns due to zirconia's unique material properties.

Clinical implication

This study provides foundational data crucial for determining the optimal design of a 4Y-PSZ double crown to achieve an appropriate retentive force.

Limitations

This study focused on evaluating single crowns; therefore, further investigations are necessary to assess the performance of multiple connected crowns. Although no fractures of the primary and secondary crowns were observed in this study, future studies should evaluate the mechanical properties based on the thicknesses of the primary and secondary crowns.

## Conclusions

This study established that higher retentive forces were achieved with increased loading pressures and smaller taper angles. Furthermore, it was indicated that repeated insertion-removal cycles led to a progressive reduction in retention. By employing advanced visual analysis to evaluate the retention mechanisms and contact conditions unique to zirconia, the findings provide foundational data supporting the clinical application of 4Y-PSZ double crowns.
